# Supplementary material for: The causal role of circulating inflammatory markers in osteoporosis: a bidirectional Mendelian randomized study
Source: Front Immunol. 2024 Jul 18;15:1412298. doi: 10.3389/fimmu.2024.1412298 (PMC11291241; doi:10.3389/fimmu.2024.1412298)
Supplement: Supplementary file 1 [file DataSheet_1.zip › supplementary material/OSTEOPOROSIS-CIM/GCST90274824.tsv.gz_leave_one_out.pdf]

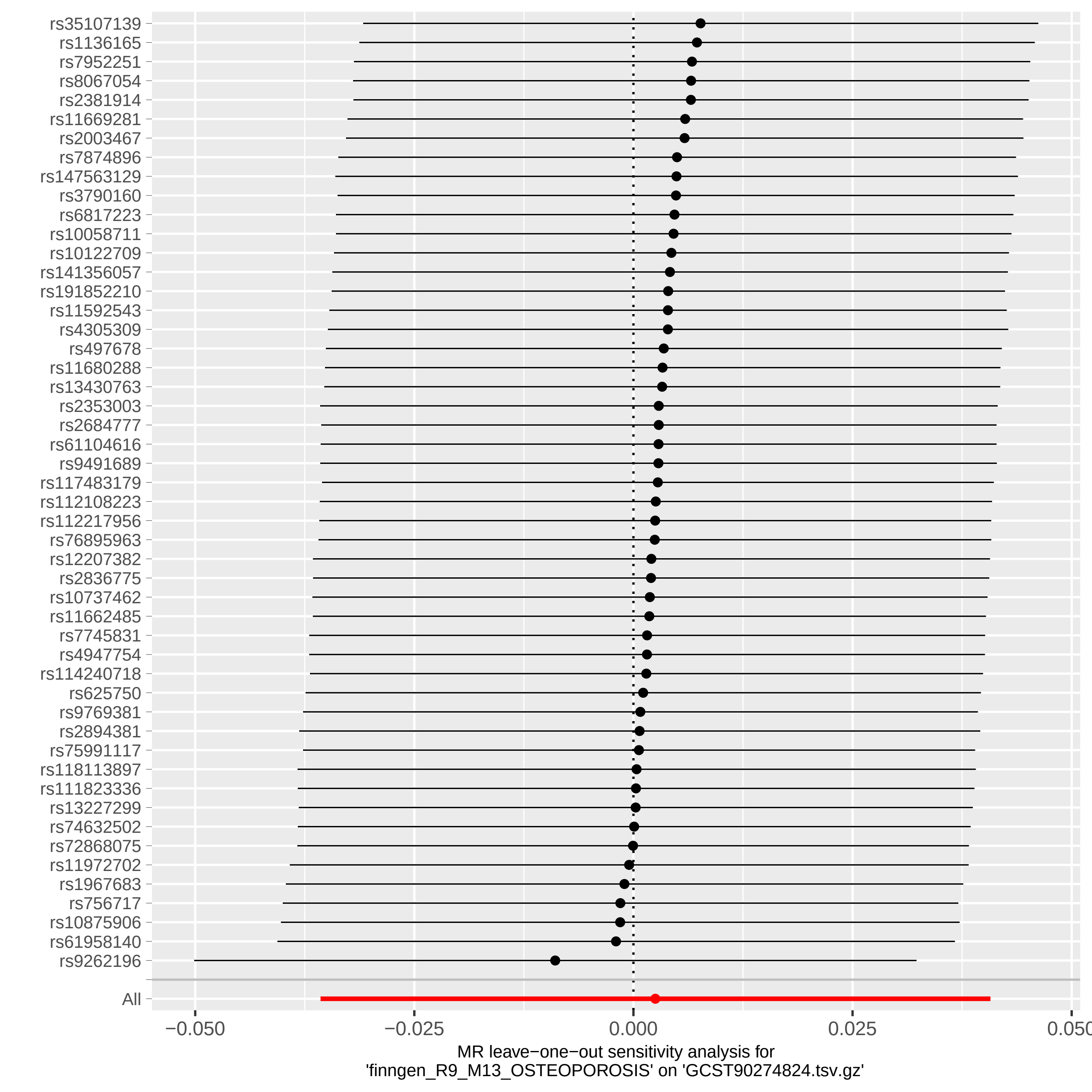
rs35107139
rs1136165
rs7952251
rs8067054
rs2381914
rs11669281
rs2003467
rs7874896
rs147563129
rs3790160
rs6817223
rs10058711
rs10122709
rs141356057
rs191852210
rs11592543
rs4305309
rs497678
rs11680288
rs13430763
rs2353003
rs2684777
rs61104616
rs9491689
rs117483179
rs112108223
rs112217956
rs76895963
rs12207382
rs2836775
rs10737462
rs11662485
rs7745831
rs4947754
rs114240718
rs625750
rs9769381
rs2894381
rs75991117
rs118113897
rs111823336
rs13227299
rs74632502
rs72868075
rs11972702
rs1967683
rs756717
rs10875906
rs61958140
rs9262196
All
−0.050
−0.025
0.000
0.025
0.050
MR leave−one−out sensitivity analysis for
'finngen_R9_M13_OSTEOPOROSIS' on 'GCST90274824.tsv.gz'
